# Overview of the Diagnostic Methods Used in the Field for Human African Trypanosomiasis: What Could Change in the Next Years?

**DOI:** 10.1155/2015/583262

**Published:** 2015-10-04

**Authors:** Julien Bonnet, Clotilde Boudot, Bertrand Courtioux

**Affiliations:** ^1^INSERM, U1094, Tropical Neuroepidemiology, Limoges, France; ^2^UMR_S 1094, Tropical Neuroepidemiology, Institute of Neuroepidemiology and Tropical Neurology, Université de Limoges, CNRS FR 3503 GEIST, 87000 Limoges, France

## Abstract

Sleeping sickness is a parasitic infection caused by two species of trypanosomes (*Trypanosoma brucei gambiense* and *rhodesiense*), transmitted by the tsetse fly. The disease eventually affects the central nervous system, resulting in severe neurological symptoms. Without treatment, death is inevitable. During the first stage of the disease, infected patients are mildly symptomatic and early detection of infection allows safer treatment (administered on an outpatient basis) which can avoid death; routine screening of the exposed population is necessary, especially in areas of high endemicity. The current therapeutic treatment of this disease, especially in stage 2, can cause complications and requires a clinical surveillance for several days. A good stage diagnosis of the disease is the cornerstone for delivering the adequate treatment. The task faced by the medical personnel is further complicated by the lack of support from local health infrastructure, which is at best weak, but often nonexistent. Therefore it is crucial to look for new more efficient technics for the diagnosis of stage which are also best suited to use in the field, in areas not possessing high-level health facilities. This review, after an overview of the disease, summarizes the current diagnosis procedures and presents the advances in the field.

## 1. General Presentation of the Disease

Human African Trypanosomiasis (HAT), or sleeping sickness, is a vector-borne parasitic disease endemic in sub-Saharan Africa. This disease is caused by an extracellular parasite called* Trypanosoma* (genus)* brucei* (species). Three subspecies exist, which possess identical morphological characteristics (presence of a flagellum, a kinetoplast, and a nucleus) but differ in their ability to infect various hosts.* Trypanosoma brucei brucei* (*T.b.b.*) is a domestic animal parasite, which transmits Nagana disease, which is not pathogenic to humans [1]. The destruction of* T. b. brucei* is caused by two trypanolytic factors (TLF) complex content in human serum. Both TLF complexes include apolipoprotein L-1 (APOL1) and haptoglobulin-related protein (Hpr). Hpr has been thought for a long time to be the active trypanolytic component of TLF. But now there are more and more confirmative evidences showing that APOL1 is the trypanolytic factor of normal human serum [2]. This parasite has proven particularly useful for research purposes. Regarding the 2 human pathogens [3],* T. b. gambiense* is an anthroponotic parasite found in 24 countries of central and western Africa and causes a chronic syndrome.* T. b. rhodesiense* is zoonotic and is endemic in 13 countries of eastern and southern Africa and causes an acute syndrome [4]. However, increasingly, the spread of* T. b. rhodesiense* has been found, especially in Uganda, where the 2 diseases forms overlap.* T. b. gambiens*e is present in the north while* T. b. rhodesiense* is present in the south, but this distribution remains artificial due to population migrations and climatic changes [5].

**Figure 1:**
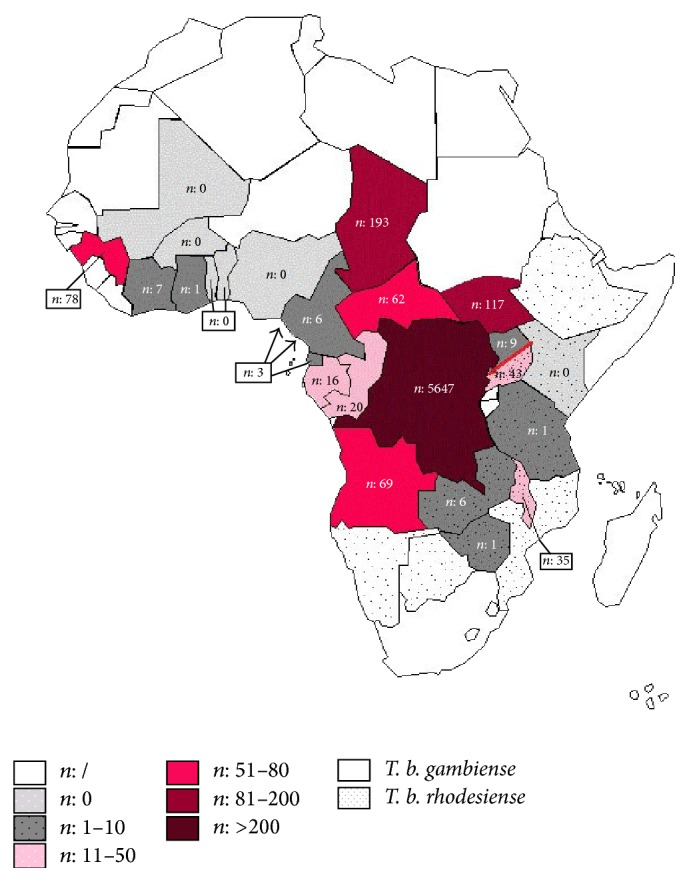
Number of new cases of HAT reported in 2013 to the WHO [1].

Recently, its prevalence has dropped, largely because of the implementation of controls and intervention programs. It belongs to the group of Neglected Tropical Diseases. Neglected Tropical Diseases are diseases that develop mainly among the poorest populations. Currently HAT is one of 17 priority Neglected Tropical Diseases recognized by WHO (World Health Organization) as Malaria, HIV, and others [6]. HAT is considered to be a huge threat to public health. Three severe epidemics have ravaged African populations. The first occurred at the end of the 19th century, the second during the 1920s, and the most recent began at the end of the 1970s and tends to be controlled today [1]. This disease outbreak is essentially linked to diverse social, economic, and political issues. Indeed, 36 sub-Saharan African countries are affected [4, 7], especially poor and remote rural regions (Figure 1). Furthermore, current estimations show that 70 million people live at risk of contracting HAT infection. Among these, 57 million people are at risk of developing* gambiense* HAT and 12.3 million people are at risk of contracting* rhodesiense* HAT [4]. This disease is considered by WHO to be one of the Neglected Tropical Diseases, for which it is necessary to establish population screening and disease control measures [4, 6].

This disease is transmitted by the bite of the tsetse fly during its blood meal. The* Glossina* vector belongs to the Diptera order.* Glossina* is viviparous and both the male and female are capable of spreading disease [1]. Many subgenus flies are involved in the transmission of parasites:* G. palpalis palpalis* and* G. p. gambiensis* transmit* T. b. gambiense* and* G. morsitans* transmits* T. b. rhodesiense* [8]. These flies need particular conditions to survive (temperature 16°C–38°C, 50%–80% relative humidity) [6]. However, the* Glossina* is classed as a “bad vector,” because it loses parasites at every blood meal, and because the female produces only 10 larvae during its lifetime [9].

**Figure 2:**
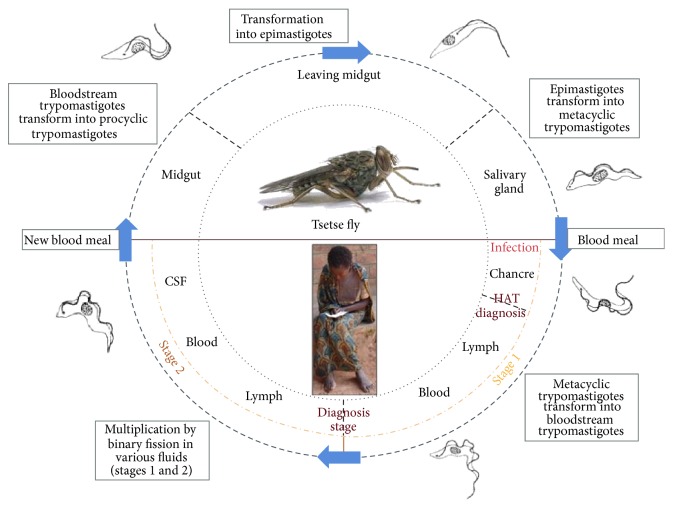
Life cycle of HAT.

During the blood meal, the infected tsetse fly injects its saliva to prevent the coagulation of the host blood, and the metacyclic trypomastigote trypanosomes are injected subdermally into the host [4]. The trypanosomes proliferate at the site of inoculation and then transform into bloodstream trypomastigotes form during the first disease stage. That form can then multiply by binary fission, in different body fluids (blood, lymph), and can move to the cerebrospinal fluid (CSF), signaling the beginning of the second disease stage. If a new, noninfected tsetse fly bites the infected host, it can ingest parasites, in their bloodstream trypomastigote form, which can move to the fly midgut, where some will differentiate into procyclic trypomastigotes. Afterwards, the parasites migrate from the midgut to the salivary gland and transform into epimastigotes. In the salivary gland, the epimastigotes further transform into metacyclic trypomastigotes and await a new fly blood meal (Figure 2).

HAT clinically evolves in two stages and the symptoms for the* T. b. gambiense* and* T. b. rhodesiense* forms are often the same, but their frequency, severity, and kinetic appearance differ. Indeed,* T. b. rhodesiense* can cause patient death within 6 months, whereas* T. b. gambiense* patients can survive for more than 10 years [10, 11].

The first stage is called the hemolymphatic or bloodstream stage and is characterized by an intermittent fever, headaches, pruritus, lymphadenopathy, asthenia, anemia, and hepatosplenomegaly [1, 4]. Once the parasites cross the blood-brain barrier (BBB), the meningoencephalic stage begins and the major symptoms are neuropsychiatric and include sleep disturbances, abnormal movement, limb paralysis, hemiparesis, irritability, aggressive behavior, and psychotic reactions [1, 4, 10]. This second stage is fatal if untreated.

Moreover, the impact on quality of life is potentially devastating, as affected subjects are unable to work for several years, which engenders poverty and social exclusion. Treatment development and therapeutic management are therefore very important. Treatments are separated into two groups.

The first group of treatments is composed of Pentamidine (Pentacarinat) and Suramin (Moranyl), and these treatments are mainly used during early disease stages. Pentamidine is the drug of choice for treatment of the* T. b. gambiense* form, while Suramin is used for* T. b. rhodesiense* treatment. However, Suramin cannot be used against* T. b. gambiense* in Western and Central Africa, because there is a risk of adverse reaction if combined with the medication used to treat* Onchocerca spp.* [1]. Pentamidine is administered intramuscularly and Suramin through intravenous injection. Pentamidine is generally well tolerated, despite side effects including hypoglycemia, nausea and vomiting, and injection site pain. Suramin can cause severe reactions, such as allergic reaction, hypersensitivity, nephrotoxicity, hematuria, or peripheral neuropathy [12].

Second stage treatments include Melarsoprol (Arsobal), Eflornithine (DFMO or *α*-Difluoromethylornithine), and the more recently developed Eflornithine/Nifurtimox combination therapy (NECT) [13]. Melarsoprol and Eflornithine are administered by intravenous injection; Nifurtimox is given orally. Melarsoprol is the only medication which can be used to treat both HAT forms [1, 12], although one of the known side effects is an increased risk of a potentially fatal encephalopathic syndrome. NECT has now become the standard first-line treatment for CNS stage* T. b. gambiense* HAT. Concerning CNS stage* T. b. rhodesiense* HAT it is intravenous Melarsoprol which is the first-line treatment [14]. However, Eflornithine causes similar adverse drug reactions as antineoplastic agents [15]. Nifurtimox can only be used in association with Eflornithine, against* T. b. gambiense*, and increases the efficacy of Eflornithine. All of these treatments require clinical surveillance during the therapeutic care. This is a major drawback for people with no access to health structures. In Table 1 are grouped the different dosages of the drugs currently used to fight against HAT.

Aside from the not insignificant adverse effects of these medications, a degree of drug resistance has evolved in the 15 to 50 years that these treatments have been employed, including Pentamidine, Melarsoprol, and Eflornithine [13, 16]. Therefore, the development of new, effective, and safe therapies is essential to advance the fight against HAT.

Recently, two new candidate drugs have been proposed. Fexinidazole, the 2-substituted 5-nitroimidazole, belongs to the nitroimidazole class of drugs [17, 18]. This pharmacological class includes many active compounds, several of which target trypanosomes. Fexinidazole was discovered in the 1980s by the Drugs for Neglected Diseases initiative (DNDi) and was developed jointly with Sanofi. In studies, Fexinidazole exhibited trypanocidal properties and demonstrated the potential to become a safe, short-course oral treatment for both HAT stages. Furthermore, this therapy, currently undergoing phase 2/3 clinical trials in treatment centers in the Democratic Republic of Congo (DRC) and Central African Republic (CAR), may avoid the necessity of disease stage screening and treatments requiring several days of hospitalization [17, 19].

The other potential treatment candidate, Benzoxaborole or SCYX-7158, is a by-product of the family of oxaboroles, developed by Anacor Pharmaceuticals. This drug proved to be highly effective in preclinical studies and is in phase 1 clinical trials today, as a single dose oral treatment for both HAT stages [18, 20]. This drug would be the ideal candidate to use for disease elimination, if current trials prove successful.

To date, these ideal treatment options are not available in the field and treatment remains “stage dependent” with serious side effects and potential complications during the second stage of the disease. Improvement in staging diagnosis and early screening methods are current challenges which would avoid delayed patient treatment.

## 2. Management of the Disease in the Field: Diagnosis

Diagnosis should be made as early as possible, in order to avoid disease progression to the neurological stage, which may necessitate complex and potentially unsafe treatments. Exhaustive screenings require major investment in personnel and material resources. In Africa such resources are often limited, especially in remote areas where the disease is most common. As a result, many infected people may die before diagnosis or treatment.

### 2.1. The Diagnosis of HAT Is Based on Active Screening (Figure 3)

**Figure 3:**
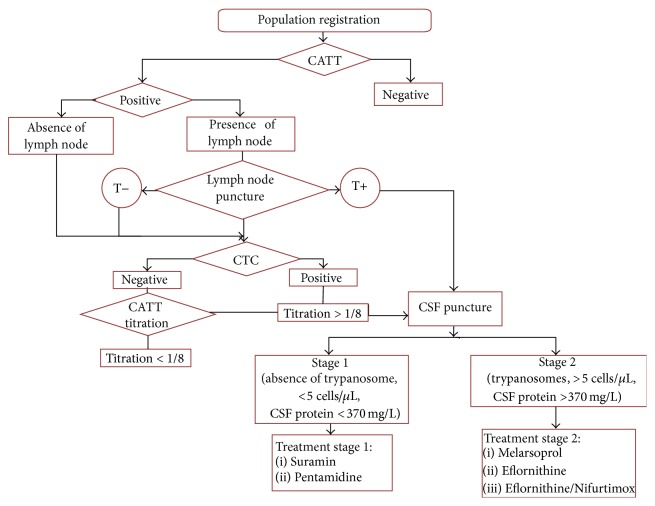
Decision tree of HAT stage diagnosis.

Antibody and parasite detection are needed for adequate patient examination and successful diagnosis in the field [1]. In this review, we only present the most currently used technics in the field and propose how to put them into practice for field diagnosis of HAT in the context of a prospective campaign with a proposition of possible decision tree (Figure 3).

#### 2.1.1. Antibody Detection

*CATT (Card-Agglutination Trypanosomiasis Test)*. CATT is a serological test, useful for initial population screening to identify suspected cases. The test was developed in the late 1970s. It can be carried out on blood, capillary blood obtained from a finger prick, or blood from impregnated filter papers [21]. Antigen used for the test CATT is complete bloodstream forms of* T. b. gambiense* variable antigen type LiTat 1.3. This test can be performed on plasma or serum dilutions for which it is more specific than the CATT on blood and is therefore used to reduce the number of false-positive reactions, often before parasitological examinations. The sensitivity of CATT on blood is about 91%, with a range of 78–99.8%, and negative predictive values as high as 99–100% have been reported in mass population screening [22, 23]. False-negative CATT results may be obtained for patients infected with strains of trypanosomes that do not express the LiTat 1.3 gene, resulting in lower sensitivity of CATT in some endemic areas [24, 25]. Despite a specificity of about 97%, the positive predictive value of the CATT remains limited when the test is used for mass screening in populations in which the overall prevalence of* gambiense* HAT is low [23, 26–28]. False-positive results are found for patients with other parasitic diseases, such as malaria and filariasis, or a transient infection with* T. b. brucei*. Parasite CATT titration is done by some control programs after all parasitological examinations. This titer also depends on the country [6]. Because of its simplicity, reliability, and low cost, it is used in all control programs for serological screening of populations at risk for* T. b. gambiense* infection.

#### 2.1.2. Parasite Detection

*(i) Lymph Node Examination*. The lymph node palpation is realized only for patient with a positive CATT. The fluid is examined rapidly after puncture. The sensitivity of lymph node palpation and aspiration varies from about 40% to 80% depending on parasite strain, stage of disease, and the prevalence of other diseases which may cause lymphadenopathy [6].

*(ii) mAECT (Mini Anion Exchange Centrifugation Technique or mAECT)*. Parasitological investigation with minicolumns by anion exchange can be carried out on venous blood. Patient blood cells are negatively charged, while trypanosomes remain neutral, so that they can be separated by anion-exchange chromatography at pH 8 [29, 30]. For mAECT, 400 *μ*L of blood is applied onto a column containing diethylaminoethyl cellulose. The blood cells stay on the gel, and the eluant containing the trypanosomes is collected in a tube. Trypanosomes are concentrated at the bottom of the tube by low-speed centrifugation (1000 g for 15 min), and the tip of the tube is examined in a special holder under a microscope (10 × 10 or ideally 10 × 16 magnification) for the presence of trypanosomes. The large blood volume used in the mAECT allows detection of fewer than 30 trypanosomes/mL, resulting in a high diagnostic sensitivity of 77% (68.8–92.1%) for mAECT [6]. This technic is time consuming and needs materials (buffer, column, etc.) and good technicians. Its use in the field is discussed by some authors and programs.

*(iii) CTC (Capillary Tube Centrifugation)*. The CTC technic is done on capillary tubes containing anticoagulant which are filled to three quarters (about 50 *μ*L) with finger-prick blood. The dry end is sealed with plasticine or by flame, avoiding heating of the blood and killing the trypanosomes. Trypanosomes are concentrated in the same layer as the white blood cells (WBCs), between the plasma and the erythrocytes, by high-speed centrifugation (12 000 g for 5 min) in a hematocrit centrifuge. The capillary tubes are mounted in a special holder or between a microscope slide and a coverslip, and the empty space between the glass surfaces is filled with water to reduce diffraction. The capillary tubes are examined at low magnification (10 × 10) for mobile parasites at the junction of the WBC layer and the plasma layer. If available, use of 16x ocular lenses facilitates recognition of trypanosomes. The detection limit of the CTC is estimated to be about 500 trypanosomes/mL. To increase its sensitivity, examination of at least four capillary tubes per person is recommended. The sensitivity is about 56% (39–80%) [6].

Disease stage identification, by examination of the cerebrospinal fluid (CSF), obtained by lumbar puncture, helps to establish the degree of progression of the disease and subsequently to determine the most appropriate treatment in each case (Figure 3).

### 2.2. Stage Diagnosis

Differentiation between the two stages can only be done by examination of the CSF after lumbar puncture. The detection of trypanosomes in CSF by microscopy alone has limited sensitivity and has a poor reproducibility rate. The number of parasites circulating in CSF can be very low, generating false negative results. An increased white blood cell (WBC) count in CSF is an indicator of meningitis and can help to increase the sensitivity of parasite detection. The WHO diagnostic criteria, which require the presence of trypanosomes in the CSF or a WBC count of more than 5 cells per *μ*L, or both [1], are the most widely used guidelines for diagnosing late stages of the disease. Some clinicians use a higher white blood cell count cutoff point such as 20 cells per *μ*L, especially for diagnosing CNS* T. b. gambiense* HAT. A consensus about the optimum WBC count of 10 cells per *μ*L has been suggested for staging HAT [13].

There are reports of some patients with CSF white blood cell count of 20 cells per *μ*L or less being treated successfully with an early-stage drug like pentamidine, which highlights the possibility of an intermediate stage of infection [31–33]. This intermediate stage is characterized by parasites which have crossed the BBB but have not yet spread to the brain parenchyma.

Another potential parameter which may assist with late-stage diagnosis is the measurement of CSF IgM concentrations, which, due to synthesis within the spinal cord, are increased early in disease development in cases where there is CNS involvement. However all of these approaches have an intrinsic drawback; there is no gold standard of CNS HAT diagnosis with which to compare any new methods [14]. Furthermore, WBC counting is not specific to sleeping sickness, and alternative diagnoses or coexisting diseases, such as malaria, syphilis, HIV infection, tuberculosis, and toxoplasmosis, need to be investigated and excluded [34–38]. Most of the articles which report on the staging of sleeping sickness disease agree that WBC counting must be supported and confirmed by newer, more advanced diagnostic procedures.

## 3. New Research Pathways to Improve the Diagnosis of HAT

### 3.1. Screening of the Population

Existing diagnostic procedures are complex and cumbersome to implement because they require specialized mobile teams, trained to carry out rapid testing using invasive protocols. Research on this disease seeks to develop simplified tests which enable the integration of activities related to HAT diagnosis within the public health infrastructure. Thus, the target of HAT phase-out by 2020, as stipulated in the WHO roadmap and the London Declaration on Neglected Tropical Diseases, will have to be achieved through the development of rapid tests which are easy to produce on a large scale [6]. Several promising tests are under development.

Lateral flow immunochromatographic devices can detect low concentrations of antibodies targeting antigens in biological fluids [39, 40]. This technology can be used to develop rapid diagnostic tests (RDTs) that detect anti-trypanosome antibodies in human finger-prick blood samples. These RDT-based lateral flow devices are simple to use and easy to read and have stability characteristics that allow wide distribution and availability in remote endemic areas. The first RDTs for HAT diagnosis are currently being tested in the field. The tests were developed by Standard Diagnostics (SD BIOLINE HAT) and Coris Bioconcept (Sero-K-SeT) [41, 42]. They are based on a device using native surface glycoproteins (VSG) LiTat 1.3 and LiTat 1.5 to test for anti-trypanosome antibodies [41, 42]. Both tests show good ranges of sensitivity and specificity when compared to CATT [42]; however, improvements are still needed especially to facilitate test production and cost. Thus, recombinant antigens are currently being produced in line with these objectives.

The second prototype device, which uses the potential ISG65 diagnosis [43], is based on a combination of recombinant and native ISG65 VSG MiTat 1.4 [44]. ISG 65 is one of two well-characterized type 1 invariant surface glycoproteins, which have moderately abundant transmembrane domains, expressed in* T. brucei* [45].

### 3.2. Advances in CSF Stage Diagnosis

The diagnosis of stage HAT is a key component in the therapeutic care of patients due to the high toxicity of some drugs including Melarsoprol that lead to arsenical encephalopathy in 5% of cases. So there is an urgent need to develop a quick, reliable, easy to perform, and cheap diagnostic test that can be used for HAT staging. The research and development of methods for disease staging have been revitalized, especially through an initiative launched by FIND and WHO in the 2000s and several alternative staging biomarkers and tools are under investigation.

#### 3.2.1. Antibodies

Many published studies have investigated disease stage diagnosis, at the molecular level. In blood, and particularly plasma samples, some studies have observed decreased levels of cytokines such as IFN-*γ* or IL-10 and NO after treatment. These markers may be compared to control subject plasma [46]. Staging studies have primarily focused on CSF as the ideal body fluid for examination, due to its proximity to the CNS. Some research has focused on CSF antibodies. An alteration in the protein concentration of CSF, such as an increase in albumin or immunoglobulin, could indicate a BBB dysfunction or increased intrathecal synthesis of proteins [47]. We have known since the 1980s that the increased concentration of immunoglobulin in the CSF and the absence of a switch between IgM and IgG are characteristic of the immune response in the brain. More recently, some publications have demonstrated that the increased intrathecal IgM fraction is a sign of the presence of a brain inflammatory process, not necessarily connected to damage of the BBB in Stage 2 HAT patients [48]. Intrathecal IgM levels are considered by many to be superior to WBC counting as a parameter for HAT staging, especially for* T. b. gambiense* cases.

#### 3.2.2. Cytokines and Chemokines

Another field of research being explored for the development of new diagnostic procedures for HAT staging is the modulation of immune-effectors such as cytokines and chemokines. The neuroinflammation seen in late stage HAT presents some characteristics such as the early activation of macrophages and astrocytes, the upregulation of inflammatory cytokines, and the presence of Mott cells (plasma cells containing IgM). Activated astrocytes and macrophages are two important sources of pro- and anti-inflammatory cytokines and chemokines in the brain. The level of these cytokines and chemokines has been measured for the investigation of their diagnostic potential both in* T. b. gambiense* and* T. b. rhodesiense*. Cytokines and chemokines are also associated with the recruitment of leukocytes to the site of inflammation and their passage through the BBB, but also with the increase of WBC observed in CSF during the second stage of HAT. The most interesting cytokines and chemokines used for staging sleeping sickness are IL-10, IL-6, IL-1*β*, CCL-3, CXCL-8, SLPI, Lipocalin 2, ICAM-1, VCAM, MMP-9, MMP-2, CXCL-10, and CXCL-13 [48, 49], which permit the activation and amplification of the immune response and allow leukocytes which are sequestered in the perivascular space to transmigrate across the basement membrane and the glial limitans to reach the brain parenchyma [50]. A recent study initially evaluated the most promising molecules such as CXL-10, CXCL-13, ICAM-1, VCAM-1, IgM, MMP-9, and B2MG and confirmed their capacity to act as accurate staging markers [50]. Furthermore, Neopterin as a new marker for staging of HAT was introduced and validated [51, 52]. Neopterin is an indicator of activation of the cellular immune response and has good potential not only as a staging marker but also for treatment outcome. The possibility of establishing a quick blood test for additional lateral disease testing, which is appropriate for field application, is advancing and is currently the primary focus of research and development [53]. This study was conducted only on* T. b. gambiense* patients. Some studies describe different outcomes for* T. b. rhodesiense* patients, largely due to the different neuropathogenesis of the two diseases [54]. In addition to being good staging markers, the level of these molecules seems to correlate with the severity of the neurological symptoms and therefore may assist with screening for the advanced second HAT stage [14]. The downside of these markers is the lack of specificity. Indeed, 80% of the CSF proteome is composed of blood derived proteins [55], and only the remaining 20% are produced in the brain, and so they are rarely considered to be specific to the neuroimmune response [47]. Moreover, these molecules are not specific markers of sleeping sickness, and other diseases such as malaria, which is also largely present in the countries affected by HAT, may also be responsible for the increased levels of these cytokines and chemokines. The vast majority of studies regarding this topic advocate the combination of multiple markers to increase staging accuracy [56, 57].

#### 3.2.3. Proteomics

Another approach currently under investigation is the evaluation of the changes in protein expression between pathological and healthy conditions. Only a few studies have established first and second stage HAT disease CSF protein profiles. Previous studies have shown a large increase in the amount of immunoglobulins for stage 2 patients [46, 48], but they also show 73 proteins which are differentially expressed between the two stages. Two of these proteins, osteopontin and beta-2-microglobulin, were confirmed to be accurate markers of first and second stage patients [58]. It is important to research and study new protein biomarkers, particularly for discriminating stage 2 and stage 1 of the disease, and this is possible thanks to progress in matters of protein and peptide analysis with the evolution of mass spectrometry, for example, [59].

#### 3.2.4. Polysomnography

In recent years, research has been conducted on the most typical clinical manifestation of HAT: the alteration of the normal sleep-wake cycle [1]. Polysomnography has been used for these studies. Polysomnography is a medical examination which involves the recording of several physiological variables, such as respiratory and heart rate, and carrying out other tests including an electroencephalogram, an electromyogram, and an electrooculogram, during patient sleep, in order to investigate sleep disorders. Studies show a high number of Sleep Onset Rapid Eye Movement Periods (SOREMP) in stage 2 patients during their sleep, not only restricted to nighttime, but also during daytime sleep too. Treatment with Melarsoprol seems to reduce the appearance of SOREMPs. In spite of the successful outcomes of these studies and the noninvasive nature of this diagnostic tool, polysomnography is largely neglected due to the difficulty in establishing the necessary environment for such examinations, which require high-tech and bulky material, trained personnel, and extended examination periods [32]. It is therefore difficult to use as a diagnostic tool in the field. Moreover, this diagnostic tool is not specific because the observed increase in SOREMPs may be attributed to other sleep disorders. In addition, SOREMPs may be detected early in the disease and so are not specific markers of stage 2 HAT.

#### 3.2.5. DNA Amplification

Carrying out PCR to amplify specific parasite DNA sequences obtained from blood, CSF, urine, or saliva samples has been proposed for staging of the disease. The loop-mediated isothermal amplification (LAMP) technic for staging HAT disease is promising and shows high specificity and sensitivity. Furthermore, for this technic, the target DNA is amplified at a constant temperature, so this test can be used in the field with minimal equipment or in the low level laboratories available in HAT endemic countries. The test can be performed on fresh blood samples, or even on blood samples which are dried on microscopy slides or on ordinary filter papers. Moreover, no gel electrophoresis is required, as positive results can be visually identified (fluorescence, white precipitate, or color change) and the analysis of several samples may be carried out simultaneously. Sets of specific primers were designed and validated and the reproducibility was verified using samples obtained from HAT patients [60–62]. This test is currently used mainly in DRC and Angola to see if there is a good candidate disease staging and is employed up to 24 months after treatment is completed, to see if it can be used to confirm disease remission (http://www.finddiagnostics.org/programs/hat-ond/hat/molecular_diagnosis.html). A similar technic for RNA amplification has recently been introduced. The Trypanozoon-specific real-time nucleic acid sequence-based amplification (NASBA) assay allows the detection of parasite 18S ribosomal RNA [63].

Recent study using CSF PCR of* T. b. gambiense* patients for stage determination indicated a good staging accuracy of PCR especially for stage 2 patients before treatment. But the presence of parasite DNA or RNA in CSF of stage 2 HAT patients should be interpreted with care as the low specificity of molecular test [63].

However, for the posttreatment follow-up, molecular biology seems to be for several authors not a good marker. The specificity and sensitivity of a diagnostic PCR mainly depends on the DNA sequence targeted by the primers. Therefore it is important to continue research for optimizing amplification, by designing new primers [64]. This technic is not quite optimal in a the field and is still debatable within scientific community.

## 4. Conclusion

After more than 50 years of neglect, the international community has recognized the need to eliminate sleeping sickness in Africa. The signing of a partnership agreement in 2001 between the WHO and Aventis announced the advent of a new era in the fight to eliminate the* Trypanosoma* parasite from the African continent. Private partners, Nongovernment Organizations (NGOs), institutional partners, and the Belgian and French governments are fully engaged, working hand in hand with relevant organizations (WHO, FAO, etc.), with the objective to find new diagnosis tests. The successful elimination of the disease in Africa needs a better management of patients. The research for new stage biomarkers for sleeping sickness is a key for the eradication of the disease since actually no dependent stage treatment is accessible by all the people concerned by HAT.

## Figures and Tables

**Table 1:** Drugs dosage used against THA.

	Pentamidine	Suramin	Melarsoprol	Eflornithine	Eflornithine/Nifurtimox
Dosage	4 mg/kg/day during 7 days	100–200 mg the first day and maximum 1 g/injection for 7 days	2.2 mg/kg/day for 10 days (for *T.b.g*) 3 × 3.6 mg/kg/day for 7 days (for *T.b.r*)	100 mg/kg/6 h during 14 days	200 mg/kg/12 h for 7 days (Eflornithine) + 5 mg/kg/3x day for 10 days (Nifurtimox)
